# Delivering eye health promotion: why and how

**Published:** 2022-03-01

**Authors:** Ada Aghaji, Clare Gilbert

**Affiliations:** 1 Senior Lecturer: College of Medicine, University of Nigeria, Enugu Campus; Consultant Ophthalmologist: Department of Ophthalmology, University of Nigeria Teaching Hospital, Enugu, Nigeria and Research Fellow: International Centre for Eye Health, London School of Hygiene & Tropical Medicine, London, UK.; 2 Professor of International Eye Health: International Centre for Eye Health, London School of Hygiene and Tropical Medicine, London, UK.

**Eye health promotion is vital for supporting the health and wellbeing of eye patients and the community, and there is a lot we can all do to help.**

**Figure:**
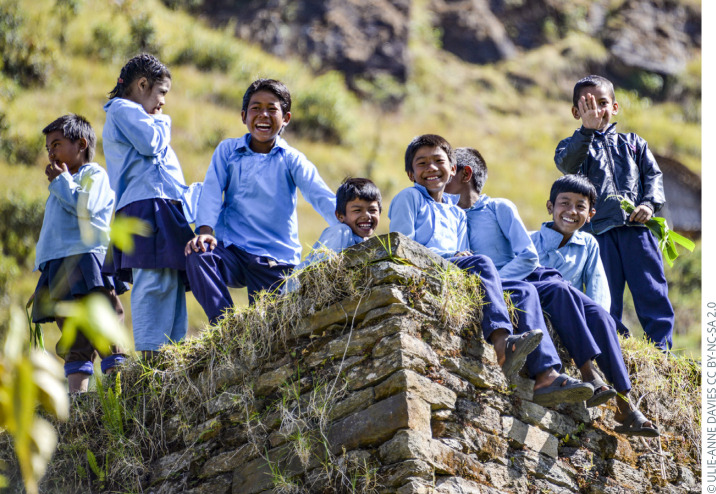
Time spent outdoors needs to be built into the school day, as this reduces the risk that children will develop myopia. **NEPAL**

In low- and/or middle-income countries, nine out of ten people who are blind or visually impaired have a condition that could have been prevented or treated.^1^ This suggests that preventive measures and eye health promotion could play an important role in reducing blindness and vision impairment. However, in most countries, little attention is paid to eye health promotion.^2^

## Why is eye health promotion needed?

Whether people remain healthy or become sick is influenced by many factors, including the conditions in which people live, their level of income, and their education. People are also more likely to become ill if they do not know how to keep themselves and their families healthy, or how and where to seek care if they become sick. The same applies to many eye conditions. For example, people are more likely to develop type 2 diabetes and vision loss from diabetic retinopathy if they do not know what causes diabetes, if they cannot afford or cook healthy food, if it is difficult for them to exercise, or if they do not understand the importance of taking their medication regularly and having their eyes examined on a regular basis. People are also more likely to become sick if there are no policies in place for affordable housing, clean water, and workplace safety, for example.

## What is health promotion?

Health promotion is the process of enabling people to increase control over, and improve, their health.^3^ Even when there is a good health system in place, three more factors are needed to ensure that people are healthy and have a good sense of wellbeing:

1. **A healthy environment** for people to live and work in.
2. **Health literacy:** knowledge and awareness of what people can do to keep themselves healthy and safe, and how and where to get help if they need it.
3. **Government policies** that support health, such as ensuring there are safe places in urban areas where people can exercise.

Health promotion is more effective when there is a focus on living standards as well as lifestyle, and when we realise that good health involves more than just the absence of disease. It is just as important for groups of people (e.g., people living with diabetes and medical experts) to work together to find relevant, workable, and acceptable solutions and to build skills so that people can bring about effective change in their own lives. These concepts and actions need to be adapted to the local setting and reach those who are hardest to reach.^4^

## Where to start

**Figure 1:**
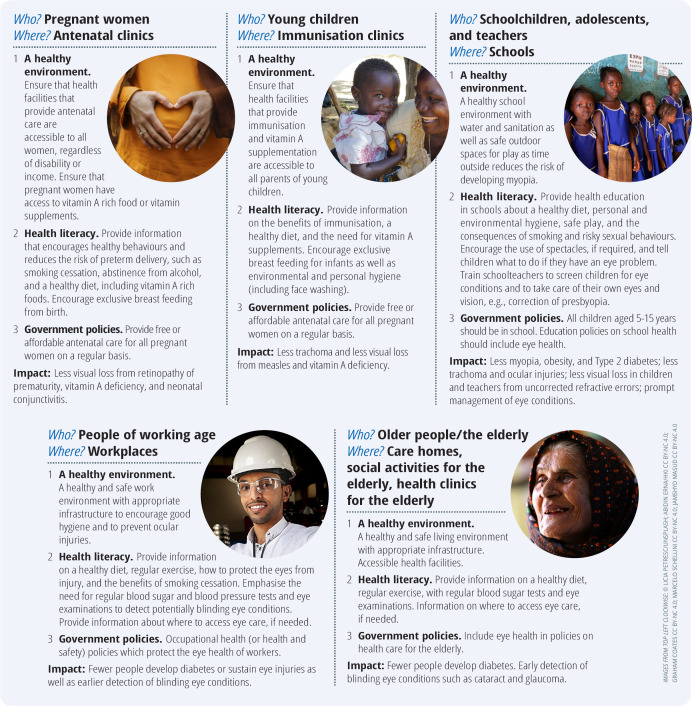
Examples of eye health promotion

A good place to start is to think about the different groups of people who will benefit from health promotion to improve their eye health (such as pregnant women, children, people with diabetes, and the elderly) and the setting where they can be reached. For each group, think about what should be in place to support their health in the three key areas of a healthy environment, health literacy, and government policies (see Figure 1).

- **A healthy environment.** What needs to be added to the environment, and what is harmful and should be removed? For example, access to water for drinking and washing is essential for a community's eye health, as is the removal of waste.
- **Health literacy**. What are the knowledge gaps and what misconceptions or false beliefs exist in the community? For example, for community members to have good eye health, they need information about diet and exercise, the importance of eye tests and blood sugar test, the benefits of cataract surgery and spectacle correction, and where to go if there is a problem with their eyes.
- **Government policies.** What policies can be put in place to support health, and which policies are potentially harmful and should be eliminated?

## What role can we play in eye health promotion?

As ophthalmologists, optometrists, ophthalmic nurses, and allied eye health personnel, we may have access to groups such as pregnant women, children, people with type 2 diabetes, and the elderly, either where we work or in the community where we live. We can train other health care workers to deliver appropriate eye health promotion. e.g., at antenatal care clinics, in diabetes clinics, or in schools. For example, eye health workers have successfully trained staff members who were working in maternal and child health clinics to deliver primary eye care, which included eye health promotion and prevention, with appreciable success.^5^ Children in school are a captive audience for eye health promotion, and the International Agency for the Prevention of Blindness has developed a school activity pack containing eye health promotional materials that can be downloaded free of charge (https://bit.ly/IAPBpack).^6^

The role of health education in eye care is to encourage the uptake of eye health promoting behaviours and increase the use of eye care services.^7^ In the course of our work, health education can be directed at individuals, families, and communities. This could include encouraging family members to have their eyes examined, particularly if there is a history of eye disease, such as glaucoma. For patients with diabetes, we could explain the importance of annual eye examinations and work with them to find ways in which they could adopt a healthier lifestyle.

“The role of health education in eye care is to encourage the uptake of eye health promoting behaviours and increase the use of eye care services.”

Improvement in the quality and quantity of services is an area of health promotion^8^ which we can also influence by making eye care services more accessible, affordable, and acceptable.

Outreach activities provide an excellent opportunity to discuss eye health promoting behaviours. We can encourage managers and regional policy makers to invest in new ways of working that prioritise health promotion. We can also advocate for policies that encourage healthy behaviours, such protective eye wear in the workplace and safe play areas in schools. World Sight Day is an excellent opportunity for eye health service providers to advocate for these policies, whether in the media, in the workplace, or in schools.

As eye health workers, we should do our best to incorporate eye health promotion into our routine eye health service delivery and become ambassadors of eye health care.
